# In vitro antifungal activities of medicinal plants used for treatment of candidiasis in Pader district, Northern Uganda

**DOI:** 10.1186/s41182-024-00628-x

**Published:** 2024-11-13

**Authors:** Betty Akwongo, Esezah K. Kakudidi, Anthony M. Nsubuga, Morgan Andama, Mary Namaganda, Patience Tugume, Savina Asiimwe, Godwin Anywar, Esther Katuura

**Affiliations:** 1 Department of Plant Science, Microbiology and Biotechnology, School of Biosciences, College of Natural Sciences, Makerere University, P. O. Box 7062, Kampala, Uganda; 2 Department of Biology, Faculty of Science, Muni University, P.O. Box 725, Arua, Uganda

**Keywords:** Medicinal plants, Antifungal activity, Candidiasis, *Candida* species, Multidrug resistance, Minimum inhibitory concentration (MIC), Minimum fungicidal concentration (MFC)

## Abstract

**Background:**

The emergence of multidrug resistant *Candida* species to available drugs has led to renewed interest in the use of herbal medicines globally. This study scientifically verified antifungal effectiveness of five commonly used plant species in Pader district, against selected pathogenic candida strains.

**Methods:**

Powdered roots of *Momordica foetida*, *Sansevieria dawei* and *Distimake dissectus*; and stem barks of *Khaya anthotheca* and *Mitragyna rubrostipulata* were extracted sequentially using petroleum ether and methanol, respectively; and total water extraction at 24.4 °C (maceration), 60 °C (decoction) and boiling water at 87 °C (hot water infusion). Extracts and their combinations, positive controls (amphotericin B, and fluconazole) and negative control (80% dimethyl sulfoxide, verified to be tolerable concentration to the tested* Candida* species) were screened and verified for their antifungal activity against *Candida albicans* (ATCC: American Type Culture Collection reference strain 10231, ATCC 90028, 0770a and 0796)*, C. glabrata* (VVc 004, ATCC 2950) and *C. tropicalis* (ATCC 750 and 0210) using agar well diffusion and broth micro-dilution, respectively.

**Results:**

Aqueous extract (24.4 °C) of *M. rubrostipulata* (ZOI: 18.00 ± 1.00 to 38.33 ± 0.17; MIC: 3.13 ± 0.00 to 20.83 ± 4.17; MFC: 12.50 ± 0.00 to 200.00 ± 0.00), methanol extract of *K. anthotheca* (10.11 ± 0.31 to 15.11 ± 0.65; 1.04 ± 0.26 to 12.50 ± 0.00; 12.50 ± 0.00 to 100.00 ± 0.00), and combination of aqueous extract (60 °C) of *D. dissectus* + methanol extract of *K. anthotheca* (7.89 ± 0.26 to 19.67 ± 0.37; 0.78 ± 0.00 to 50.00 ± 0.00; 12.50 ± 0.00 to 200.00 ± 0.00) exhibited broad spectrum antifungal activities and were fungistatic against all tested* Candida* species, which comprised 8 clinical/control and susceptible/resistant strains. None of the conventional drugs used demonstrated broad spectrum antifungal activity across all tested* Candida* species/strains.

**Conclusion:**

Methanol extract of *K. anthotheca*, aqueous extract (24.4 °C) of *M. rubrostipulata,* and combination of aqueous extract (60 °C) of *D. dissectus* + methanol extract of *K. anthotheca* could be effective in the treatment of candidiasis. They demonstrated potential broad spectrum antifungal activity against different species and strains of tested Candida than the fluconazole and amphotericin B drugs. Their fungistatic nature showed their ability to inhibit fungal growth. Hence, these extracts/extract combination can offer better treatment option for candidiasis if they are standardized and also their active curative compounds isolated and made into antifungal drugs.

## Introduction

Globally it is estimated that, 1.5 million people die from invasive fungal infections annually [1]. In most African countries, many people are affected with oropharyngeal and vulvovaginal candidiasis due to mostly their immuno-compromised status [2]. In Uganda, the situation is not different since 4,099,357 people (about 9% of the total population) get fungal infections [3] and about 38,000 people die yearly, mainly from HIV-related fungal infections [2]. Most of these cases are from Eastern and Northern Uganda where Pader district is located, these regions contain the highest burden of HIV-related opportunistic infections in the country, especially oral candida [4]. The current treatment of candidiasis involves the use of orthodox medicines, and fluconazole is widely used antifungal drug of choice for first-line prophylaxis treatment of mainly candidiasis [5, 6]. Similarly in Uganda, serious fungal infections of Candida like *Candida vaginitis* is also treated with fluconazole [3]. However the antifungal drugs are scarce and expensive [7], and most fungi have developed resistance to them [8]. This can be partly attributed to the small number of antifungal drug classes [9]. The available antifungal drugs are also expensive and not easily accessible especially to the poor communities [6]. These factors have resulted in the resurgence in medicinal plants use globally. Thus traditional medicine meets the primary health care needs to about 80% of people in the developing countries [10]. Many plants have been documented for treatment of various fungal infections, and some of the plants like *Punica granatum* L., *Eucalyptus globulus* Labill, *Artemisia mexicana* Willd. among others, have demonstrated broad antifungal potential including anti-candida activity [11, 12]. Medicinal plants of *Momordica foetida* Schumach., *Sansevieria dawei* Stapf, *Khaya anthotheca* (Welw.) C.DC., *Distimake dissectus* (Jacq.) A. R. Simões & Staples and *Mitragyna rubrostipulata* (K.Schum.) Havil. are widely used by communities of Pader district in Northern Uganda for treatment of both oropharyngeal candidiasis (OPC) and vulvovaginal candidiasis (VVC) [13].

The high fungal disease burden therefore poses a serious health challenge and requires a multi-pronged approach to deal with it. Medicinal plants offer viable prospects because of the relatively high success rates of drug discovery from medicinal plant compared to other methods [14]. This study thus sought to validate effectiveness of medicinal plant species used by the community in Pader district against* Candida* species, as a potential source of antifungal drugs. This aligns with SDG 3 of promoting good health and well-being for all by 2030.

## Materials and methods

### Plant materials

The root and stem barks of five of the most cited medicinal plants, based on their high Informant Consensus Factor (FIC) values for the treatment of candidiasis by the communities of Pader district, from an earlier ethnobotanical study by Akwongo et al. [13] were tested in this study as shown in Table 1.

**Table 1:** Plant species/parts tested against *Candida* spp in this study

	Plant scientific names and voucher number	Part used	GPS coordinates of plant species collection sites
1	*Momordica foetida* Schumach BA005	Root	03°0′25″ N 33°0′56″ E; Altitude 1051.1 m
2	*Sansevieria dawei* Stapf BA002	Root	03°0′26″ N 33°0′50″ E; Altitude 1072.9 m
3	*Khaya anthotheca* (Welw.) C.DC BA013	Stem bark	03°0′22’’N 33°1′43″E; Altitude 1080.9 m
4	*Distimake dissectus* (Jacq.) A. R. Simões & Staples BA032	Root	02°54′49″N 32°55′56″E; Altitude 1052.3 m
5	*Mitragyna rubrostipulata* (K.Schum.) Havil. (Earlier known as *Hallea rubrostipulata* (K. Schum.) Leroy BA019	Stem bark	02°54′49″N 32°55′56″E; Altitude 1052.3 m

The plants were collected in June 2022 from Lapul and Pajule sub-counties in Pader district (32°45′E–33°00′E and 2°45′N–3°00′N). Voucher specimens of the collected plant species were identified at Makerere University Herbarium (MHU). We reported *Distimake dissectus* (Jacq.) A. R. Simões & Staples, for the first time in Uganda and deposited the first voucher specimen at the MHU, after it was jointly identified by taxonomists Dr. Mary Namaganda and Dr. Eunice Olet. The names of the identified plant species were verified using Plants of the World (POWO).

### Plant extracts preparation

The dried plant materials were powdered and used to prepare extracts using petroleum ether and methanol solvents. The assay was done according to Vaghasiya and Chanda [15], with slight modifications of using solvents of increasing polarities viz. petroleum ether (non-polar solvent) followed by methanol (polar solvent). In the same way, 100 g of powered plant samples was used to prepare aqueous extracts using decoction (at 60 °C) [16], maceration with boiled cooled distilled water at room temperature, 24.4 °C [17] and hot water infusion (87 °C) [17]. All dry extracts were labelled and weighed to obtain the percentage extraction yields using the equation of Aruwa et al. [18]:

1 $${\text{Extract yield}}\, = \,\left( {{\text{weight of dried extract}}/{\text{weight of the dried plant samples}}} \right) \times {1}00\% .$$

The dried extract powders were placed in stoppered sample vials, and maintained at 4 °C, until their use for antifungal activity determination.

### Antifungal activity tests

#### Antifungal organisms

***Candida*** **species/strains**

The quality control fungal strains, viz C. *albicans* ATCC 10231, *C. albicans* ATCC 90028, *C. tropicalis* ATCC 750 and *C. glabrata* ATCC 2950 were supplied as culti-loops (Thermo scientific, USA). Clinical fungal isolates (*C. albicans* 0796, *C. albicans* 0770a and *C. tropicalis* 0210), whose identities and in vitro antifungal susceptibilities were already determined by automated VITEK 2 (bioMérieux) compact system (card AST-YS08) were obtained as trial vials from Kiruddu National Referral Hospital, Kampala. *Candida glabrata* VVc 004 was obtained from Mbarara University of Science and Technology as *Candida albicans* VVc004. It was verified using automated VITEK 2 at Kiruddu National Referral Hospital, which showed that it was *Candida glabrata* susceptible to fluconazole, and thus was used as *C. glabrata* VVc 004 for this study. The species and strains of candida were all maintained at 4 °C.* Candida* species/strains and their susceptibilities to the conventional drugs are summarized in Table 2.

**Table 2:** *Candida* species/strains and their susceptibilities to the conventional drugs

*Candida* species/strains							
*Candida albicans*				Non-*Candida albicans*			
Resistant to fluconazole		Susceptible to fluconazole		Susceptible to fluconazole			
*C. albicans ATCC 10231*	*C. albicans 0796*	*C. albicans ATCC 90028*	*C. albicans 0770a*	*C. glabrata ATCC 2950*	*C. glabrata VVc* 004	*C. tropicalis ATCC 750*	*C. tropicalis 0210*

The quality control strains are in reference to fluconazole; all clinical isolates are susceptible to Amphotericin B

Prior to the experiment, the* Candida* species were resuscitated in Brain Heart Infusion Broth (BHIB) (Oxoid) which was prepared following the manufacturer’s instructions (Thermo Scientific, Oxoid) (https://labmal.com/product/brain-heart-infusion-broth-500g/). The loops containing different quality control* Candida* species (ATCC) were each placed in separate glass tubes of BHIB. For clinical isolates, 100 µl of each of the culture suspensions were dispensed into separate BHIB media in glass tubes using micro-titre pipette. All the glass tubes with their contents were incubated at 37 °C for 24 h. Thereafter, growth of fungal cells was observed by turbidity of the media in the glass tubes. Culture broths were inoculated on Sabouraud dextrose agar (SDA) that was freshly prepared according to the manufacturer’s instructions (Oxoid, United Kingdom), and then acidified with 1% lactic acid to impede any kind of bacterial growth. The inoculated SDA were incubated aerobically at 37 °C for 24 h [19]. The grown yeast cells were passaged on SDA to ensure purity and viability before inoculum preparation, and were maintained at 4 °C.

**Inoculum preparation**

The inoculum was prepared according to Clinical Laboratory Institute CLSI M27-A3 [20] with modifications of incubation temperature to 37 °C for better growth of yeast according to EUCAST [21], and fungal growth time to 24–48 h, to attain the required colony diameters recommended by CLSI M27- A3 [20], which is ≥ 1 mm [19]. Five colonies of ≥ 1 mm in diameter were picked from 24 to 48-h-old cultures and suspended in 5 mL of sterile 0.145-mol/L saline (8.5 g/L NaCl; 0.85% saline). The resulting suspension was vortexed for 15 s, and the cell turbidity adjusted with a spectrophotometer at 530 nm by adding sufficient sterile saline to increase the transmittance to that produced by a 0.5 McFarland of 1 × 10^6^ to 5 × 10^6^ cells per mL. This cell count produces confluent growth of* Candida* species on the agar plate, thus, was used for zone of inhibition determination [22].

For minimum inhibitory concentration (MIC) determination, a working suspension was made by a 1:100 dilution followed by a 1:20 dilution of the stock suspension with RPMI 1640 broth medium buffered to pH 6.9–7.1 containing 3-(N-morpholino) propanesulfonic acid [MOPS] at a concentration of 0.164 mol/l (34.53 g/l), which resulted in 0.5 × 10^3^ to 2.5 × 10^3^ cfu/ml.

#### Antifungal assays

**Preparation of positive controls**

The conventional antifungal drug powder, fluconazole analytical grade (Sigma-Aldrich, India), fluconazole pharmaceutical grade (Pfizer, France) and Amphotericin B (Gilead sciences international Ltd, Cambridge) were used as positive controls. Stock solutions at concentration of 1280 µg/mL were prepared, using sterile distilled water [23]. For MIC determination, the prepared solutions were diluted in Roswell Park Memorial Institute (RPMI) medium to give test concentrations ranging from 64 to 0.125 µg/mL, which encompasses breakpoint concentrations for MIC testing based on current antifungal clinical breakpoints and interpretive categories for yeasts for broth microdilution (fluconazole: S: ≤ 2 µg/mL, SDD: 4–32 µg/mL and R: 8–64 µg/mL) [20, 22].

**Preparation of stock solutions of plant extracts**

Eighty percent dimethyl sulfoxide (DMSO) (GC grade, Spectrochem, India) was used for complete dissolution of all the plant extracts for this study (aqueous, methanol and petroleum ether), having been first tested and found to be tolerable concentration to all the selected strains of *Candida albicans*, *Candida tropicalis* and *Candida glabrata* species, with zero zone of inhibitions from Agar well diffusion tests (Table 5). Use of 80% DMSO as a diluent was a modification of Nejad et al. [24]; Wenji et al. [25] and Ruiz-Duran et al. [26] protocols who used 100% DMSO as safe extract diluent against similar tested *Candida* species. Stock concentration of 400 mg/ml was made for screening of the plant extracts for antifungal activity [27].

**Agar well diffusion for antifungal activity screening of single-plant extracts**

The yeast suspension of 100 μl (1 × 10^6^ to 5 × 10^6^ cells per mL) were pipetted on the surface of Mueller–Hinton Agar medium (MHA) (India) that was not supplemented with glucose to avoid fungal overgrowth, and non-supplemented with methylene blue dye due to clear inhibition zone edge demarcations [28]. The suspensions were surface spread over the entire agar surface a total of 3 times, with rotation of the plate approximately 60° each time to ensure even distribution of the inoculum. The inoculation was done within 15 min of inoculum suspension preparation to avoid change of its density [29]. The swabbed Mueller–Hinton Agar plates stood for 15 min to allow the attachment of yeast on the media [22, 27].

Wells of diameter of 6 mm each were punched aseptically with a sterile cork borer into the agar. Thereafter, 50 µL of 400 mg/ml of each extract stock solutions, were pipetted using a fixed-volume micropipette and introduced into the wells. Plates with wells filled with 50 µL fluconazole analytical grade, fluconazole pharmaceutical grade and Amphotericin B (6.4 μg) separately; and 50 µL of 80% DMSO were the positive and negative controls, respectively [30]. The inoculated plates stood in the biosafety cabinet for 2 h to facilitate diffusion of applied solutions in the media. Subsequently, the Petri dishes were incubated at 35 ± 2 °C for 24 h. The experiments were replicated and read at 24 hourly intervals for 3 days, i.e. 24 h, 48 h and 72 h (n = 9) to increase reliability. Measurement of the diameters of the zones of inhibition around the wells were done using a mathematical divider and a transparent ruler in millimetres [27]. The extent of antifungal activity of the test plant extracts were interpreted according to Arenas et al. [31] with slight modifications of interpretive ranges of antifungal activity intensity, to include all plants with zone of inhibition (ZOI) to have exhibited antifungal activity to some extent, recorded as: +  = very low activity (ZOI: < 10 mm); +  +  = low activity (ZOI: 10–13 mm); +  +  +  = moderate activity (ZOI: 14–19 mm ZOI), +  +  +  +  = very high activity (ZOI: > 19 mm).

**Agar well diffusion/antifungal activity screening of combinations of plant extracts with demonstrated antifungal activity**

Antifungal activity of plant extract combinations were to verify antifungal potentials of plant combinations used traditionally for treatment of candidiasis [13]. This is because synergistic antimicrobial combinations are known to offer therapeutic remedies for antimicrobial resistance [32]. The synergistic antimicrobial potentials were determined only for the active plant extracts, by making intra- and inter-extract combinations of the active plant species [33]. The plant extract combinations were constituted as shown in Table 3.

**Table 3:** Intra- and inter-extract combinations of active plant species

Plant species	Extracts combinations	Vol of DMSO used (mls)
1. *K. anthotheca (Ka)*	*0.4 g Ka* aqueous^1^** + ***0.4 g Ka* methanol	2
2. *M. rubrostipulata (Mr)*	0.4 g *Mr* aqueous^1^** + ***0.4 g Mr* methanol	2
	0.4 g *Mr* aqueous^1^** + **0.4 g *Mr* aqueous^3^	2
	*0.4 g Mr* methanol + 0.4 g *Mr* aqueous^3^	2
	*0.2 g Mr* methanol + 0.2 g *Mr* aqueous^3^ + 0.4 g *Mr* aqueous^1^	2
	0.4 g *Mr* aqueous^1^ + *0.4 g Mr* methanol + 0.4 g *Mr* aqueous^3^	3
3. *D. dissectus (Dd)* + *M. rubrostipulata (Mr)*	0.4 g *Dd* aqueous^2^ + 0.4 g *Mr* aqueous^1^	2
4. *D. dissectus (Dd)* + *K. anthotheca (Ka)*	0.4 g *Dd* aqueous^2^ + 0.4 g *Ka* aqueous^1^	2
5. *K. anthotheca (Ka)* + *M. rubrostipulata (Mr)*	0.4 g *Ka* aqueous^1^ + 0.4 g *Mr* aqueous^1^	2
6. *D. dissectus (Dd)* + *K. anthotheca (Ka)* + *M. rubrostipulata (Mr)*	0.4 g *Dd* aqueous^2^ + 0.4 g *Ka* aqueous^1^ + 0.4 g *Mr* aqueous^1^	3
7. *M. rubrostipulata (Mr)* + *K. anthotheca (Ka)*	0.4 g *Mr* aqueous^1^ + *0.4 g Ka* methanol	2
8. *D. dissectus (Dd)* + *K. anthotheca (Ka)*	0.4 g *Dd* aqueous^2^ + *0.4 g Ka* methanol	2
9. *D. dissectus (Dd)* + *K. anthotheca (Ka)* + *M. rubrostipulata (Mr)*	0.4 g *Dd* aqueous^2^ + *0.4 g Ka* methanol + 0.4 g *Mr* aqueous^1^	3

Key: Aqueous^1^ =  cold water at 24.4 °C; aqueous^2^ =  hot water at 60 °C; aqueous^3^ =  hot water at 87 °C; Vol =  volume

**Antifungal activity of extracts combinations**

The extracts combinations were screened for antifungal activity using agar well diffusion assay as described previously in Section "Agar well diffusion for antifungal activity screening of single plant extracts".

#### Determination of minimum inhibitory concentration (MIC)

**Fungal growth medium**

Synthetic RPMI medium 1640 (X1, UK) [with glutamine, without bicarbonate, and with a pH indicator; supplemented with 2% glucose, buffered with 3-(N-morpholino) propane sulfonic acid (MOPs) at a concentration of 0.164 mol/l (34.53 g/l), pH 6.9–7] was used to determine MIC of plant extracts/drugs [20, 34]. This is because RPMI medium 1640 is a superior medium that results in lower MICs than when other media are used [35].

**Supplementing RPMI (X1) with 2% glucose**

The glucose concentration in RPMI 1640 (1X) was adjusted from 0.2% to 2% as recommended in EUCAST [21], by adding 1.8% of extra pure glucose. The pH was maintained at a range of 6.9–7.1 using 10 M MOPs sodium salt as the buffer by making a final concentration of 0.165 M in the medium. Briefly, 500 ml of the RPMI 1640 medium was supplemented with 2% glucose by adding 90ml of a 10% sterilized glucose solution and 8.25 ml of the 10 M MOPS to 401.75 ml of RPMI medium. This made a final volume of 500 ml of 2% RPMI 1640 medium. Addition of MOPs raised the pH of RPMI 1640 medium to 8.43. Final pH of 7.08 (6.9–7.1 range) was achieved by adding 2M HCl drop-wise (each drop contained 10 μl) and monitored using a pH meter.

*Minimum inhibitory concentration (MIC) of antifungal agents*

The MIC was determined for all the plant extracts and extract combinations that showed antifungal activity in the agar well diffusion. Exactly 100 µL medium of RPMI 1640 was filled in sterile microplates (96 U-shaped wells) from 1 to 12 wells [20]. Then 100 μl of plant extracts (400 mg/ml / 200 mg/ml / 100 mg/ml) were added to the first 1–7 wells in the first row of the microplates using a micropipette. The extracts were subsequently serially double diluted down the columns using a multi-channel micropipette. The contents of the first wells were thoroughly mixed by rinsing 5 times using a multi-channel micropipette. After that, 100 μl of the mixture were pipetted and then transferred to the second wells, and mixed thoroughly. Consequently, 100 μl of the contents of the second wells were transferred to the third wells using new micropipette tips after thoroughly mixing. This procedure was repeated up to the eighth wells, where 100 μl of the thoroughly mixed content was pipetted and discarded so as to maintain the same volume of fluid in all the wells (EUCAST, 2003; 28). The above serial double dilutions from wells 1–7 containing 400 mg/ml plant extracts produced concentrations of 200, 100, 50, 25, 12.5, 6.25, 3.125 and 1.5625 mg/ml; wells containing 200 mg/ml extracts due to strong antifungal activity, produced concentrations of 100, 50, 25, 12.5, 6.25, 3.125, 1.5625 and 0.78125 mg/ml; and wells with 100 mg/ml plant extracts resulted in 50, 25, 12.5, 6.25, 3.125,1.5625, 0.78125 and 0.390625 mg/ml correspondingly [27]. For the positive controls; 100 µL of 64 µg/mL (0.064 mg/ml) fluconazole analytical grade was added to well 8, 100 µL of 64 µg/mL (0.064 mg/ml) fluconazole laboratory grade was added to well 9, and 100 µL of 64 µg/mL (0.064 mg/ml) amphotericin B analytical grade was added to well 10. The fluconazole and amphotericin B drugs (positive controls) were serially double diluted following the same procedures for diluting plant extracts above, that resulted in drug concentrations of 32, 8, 4, 2, 1, 0.5, 0.25 and 0.125 µg/mL from wells 8–10, respectively. Wells 11 and 12 contained the added 100 µL of medium for growth and sterility controls (the blank) [23]. Exactly 100 µL of diluted inoculum suspension (0.5 × 10^3^ to 2.5 × 10^3^ cfu/ml) was pipetted and first added to well 11 (growth control), then subsequently to the tenth, until well number one. This reduced the error of contaminating the growth control well. Exactly 100 µL of the RPMI medium was pipetted to well 12. The microdilution plates were covered with their lids/aluminium foil and ten incubated at 37 °C [21] for 24–48 h [23].

Since the plant extracts were turbid/coloured, the MIC of the plant extracts/drugs on candida strains were observed visually for any reduction in resazurin dye by change of colour from blue colour to pink [27, 36] or brown/colourless caused by long/overnight incubation due to variation in resazurin reduction time of the cell line under investigation [37, 38]. Colour change in the resazurin dye was an indicator of metabolic activity of active fungal cells in the wells [36]. The lowest plant extracts/drug concentrations that inhibited visible growth of the tested* Candida* species/strains/highest dilutions were chosen as the MIC values (lowest drug/extract dilution X original plant extract concentration). The methods described by Ohikhena et al. [36] was used, in which 20 μL of 0.01% resazurin was added to all the wells (from the 12th to the 1st well) and was further incubated for overnight at 37 °C. The growth in each well were compared with the growth in the control/drug-free wells. Triplicates of the experimentation were done.

The susceptibility of potential antifungal plants to *Candida* species was based on the lowest values of MIC attained. The MICs for antifungal drugs: fluconazole were interpreted following CLSI M27-A3 guidelines [20]: susceptible (S): ≤ 2 µg/mL, susceptible dose dependent (SDD): 4–32 µg/mL and resistant (R): 8–64 µg/mL) [22]; while amphotericin B susceptibilities were interpreted based on CLSI M27-A2 guidelines [23] (susceptible (S): ≤ 1 µg/mL and resistant (R): > 1 µg/mL).

#### Determination of minimum fungicidal concentrations (MFCs)

The MFCs were determined for all the wells that did not show fungal growth from MIC results to ascertain if the extracts/drugs were fungicidal. The contents of these wells were homogenized with micropipette tips, and an aliquot from each well [30] was sub-cultured by streaking on SDA containing 1% lactic acid to suppress bacterial growth [39]. The lowest concentration of the extracts/drugs that did not permit any fungal colony growths after incubation for 18–24 h at 37 °C were taken as MFC [40]. The MFCs were calculated as lowest drug/extract dilution X original plant extract concentration. Duplicates of experiments were done.

### Statistical analysis

Descriptive statistics (mean and standard error of the mean) were computed for weights of the plant extracts, zone of inhibition (ZOI), MICs and MFCs using SPSS version 20. Mean values for weights of the plant extracts, zone of inhibition (ZOI), MICs and MFCs were expressed as mean ± SEM. Mean comparisons of ZOI of different extracts, conventional drugs (positive controls) and DMSO (negative control) were performed by one-way ANOVA followed by Tukey’s HSD post hoc multiple comparison test.

## Results

### Plant extracts yields

The percentage extracts yields of each of the five plant species significantly differed across each of the solvents used (*p* < 0.05), from highest to lowest extracts yields were; aq. extract at 87 °C, methanol extract, aq. extract at room temperature (24.4 °C), aq. extract at 60 °C and petroleum ether extract (Table 4).

**Table 4:** Extract yields (%) (mean ± SE) of plant species in different solvents (n = 3)

Plant species	Solvents					ANOVA	
	Petroleum ether	Methanol	Aqueous^1^	Aqueous^2^	Aqueous^3^	F	p
1. *M. foetida*	0.27 ± 0.01	9.31 ± 0.01	4.96 ± 0.01	6.83 ± 0.01	19.79 ± 0.01	3388941.79	0.00
2. *S. dawei*	0.22 ± 0.01	15.30 ± 0.01	18.60 ± 0.01	15.53 ± 0.01	27.15 ± 0.01	546166.75	0.00
3. *K. anthotheca*	0.33 ± 0.01	16.13 ± 0.01	9.36 ± 0.01	11.08 ± 0.01	26.47 ± 0.01	1657758.44	0.00
4. *M. rubrostipulata*	2.59 ± 0.01	20.67 ± 0.01	6.70 ± 0.01	7.38 ± 0.01	5.90 ± 0.01	251021.36	0.00
5. *D. dissectus*	0.06 ± 0.01	5.98 ± 0.01	5.03 ± 0.01	2.58 ± 0.01	25.05 ± 0.01	556368.30	0.00
F	21235.27	1525071.10	2085116.29	1187733.72	155402.74		
p	0.00	0.00	0.00	0.00	0.00		

Key: Aqueous^1^ =  cold water at 24.4 °C; Aqueous^2^ =  hot water at 60 °C; aqueous^3^ =  hot water at 87 °C

### Antifungal activity

#### Preliminary screening of antifungal susceptibility of selected *Candida* species to five potential antifungal plant species

All the organisms including; *C. albicans,* *C. glabrata* and *C. tropicalis* tested in this study demonstrated antifungal susceptibility to methanol extract of *K. anthotheca*, and aq. extract (24.4 °C) of *M. rubrostipulata*. However, none of the* Candida* species were susceptible to any of the extracts of *M. foetida* and *S. dawei* and to any of the petroleum extracts of all the five plant species (Table 5). Aqueous extract (24.4 °C) of *M. rubrostipulata* had the greatest extent of inhibition of candida growth to most of the tested* Candida* species, with high activity/growth inhibition (+ +  + +) recorded against *C. albicans* ATCC 90028, *C. glabrata* ATCC 2950, *C. tropicalis* ATCC 750 and *C. tropicalis* 0210; and moderate activity (+ + +) against *C. albicans* ATCC 10231, *C. albicans* 0770a and *C. albicans* 0796. The methanol extract of *K. anthotheca* showed the second highest extent of inhibition of candida growth to most of the tested* Candida* species, with moderate activity/growth inhibition (+ + +) recorded against *C. albicans* ATCC 10231, *C. albicans* ATCC 90028 and *C. albicans* 0770a; and low activity (+ +) against *C. albicans* 0796, *C. glabrata* ATCC 2950, *C. tropicalis* ATCC 750 and *C. tropicalis* 0210 (Table 5).

**Table 5:** Antifungal susceptibility of *C. albicans, C. glabrata and C. tropicalis* to five potential antifungal plants

Plant species	Extract categories	*Candida albicans*				*Candida glabrata *and *Candida tropicalis*			
		Resistant to fluconazole		Susceptible to fluconazole		Susceptible to fluconazole			
		*C. albicans* ATCC 10231	*C. albicans* 0796	*C. albicans* ATCC 90028	*C. albicans* 0770a	*C. glabrata* ATCC 2950	*C. glabrata* VVc 004	*C. tropicalis* ATCC 750	*C. tropicalis* 0210
1. *M. foetida*	Aqueous^1^	−	−	−	−	−	−	−	−
	Aqueous^2^	−	−	−	−	−	−	−	−
	Aqueous^3^	−	−	−	−	−	−	−	−
	Petroleum ether	−	−	−	−	−	−	−	−
	Methanol	−	−	−	−	−	−	−	−
2. *K. anthotheca*	Aqueous^1^	+ +	+ +	+ + +	+ +	−	−	+ + + +	+
	Aqueous^2^	−	−	−	−	−	−	−	−
	Aqueous^3^	−	−	−	−	−	−	−	−
	Petroleum ether	−	−	−	−	−	−	−	−
	**Methanol**	** + + + **	** + + **	** + + + **	** + + + **	** + + **	** + + **	** + + **	** + + **
*3. S. dawei*	Aqueous^1^	−	−	−	−	−	−	−	−
	Aqueous^2^	−	−	−	−	−	−	−	−
	Aqueous^3^	−	−	−	−	−	−	−	−
	Petroleum ether	−	−	−	−	−	−	−	−
	Methanol	−	−	−	−	−	−	−	−
*4. D. dissectus*	Aqueous^1^	−	−	−	−	−	−	−	−
	Aqueous^2^	+ +	+ +	+ +	+ +	ˉ	+	+ + +	+
	Aqueous^3^	−	−	−	−	−	−	−	−
	Petroleum ether	−	−	−	−	−	−	−	−
	Methanol	−	−	−	−	−	−	−	−
*5. M. rubrostipulata*	**Aqueous**^**1**^	** + + + **	** + + + **	** + + + + **	** + + + **	** + + + + **	** + + + **	** + + + + **	** + + + + **
	Aqueous^2^	−	−	−	−	−	−	−	−
	Aqueous^3^	+	+	+	+	+ + +	−	+ + +	−
	Petroleum ether	−	−	−	−	−	−	−	−
	Methanol	+ +	+ +	+ +	+ +	−	−	+	−

Key: Aqueous^1 ^= cold water at 24.4 °C; Aqueous^2^ =  hot water at 60 °C; Aqueous^3^ =  hot water at 87 °C

Zone of candida growth inhibition (ZOI) −= no activity (ZOI: < 07 mm); +  = very low activity (ZOI: 07 < 10 mm); +  +  = low activity (ZOI: 10–13 mm); +  +  +  = moderate activity (ZOI: 14–19 mm ZOI); +  +  + +  = very active (ZOI: > 19 mm)

#### Zones of inhibition (ZOI) of the antimicrobial agents against selected *C. albicans*, *C. glabrata and C. tropicalis* species

**Susceptibility of** ***Candida albicans*** **species to single-plant extracts, extracts combinations and conventional drugs**

All the susceptible *C. albicans* (ATCC 90028, 0770a) and resistant *C. albicans* (ATCC 10231, 0796) were susceptible to a total of 10 plant extracts including extracts combinations, and amphotericin B; of which aq. extract (24.4 °C) of *M. rubrostipulata* (ZOI: 18.00 ± 1.00 to 20.56 ± 0.56) and amphotericin B drug (ZOI: 13.42 ± 0.54 to 22.32 ± 0.32) demonstrated significant highest zones of inhibition, respectively, across *C. albicans* species. The other extracts and extract combinations with moderate ZOIs in decreasing order include; combination of aq. extract (60 °C) of *D. dissectus* + methanol extract of *K. anthotheca* and methanol extract of *K. anthotheca*, respectively, among other extracts as shown in Table 6. All the four *C. albicans* strains were not susceptible to both fluconazole analytical and pharmaceutical grades (Table 6), which justified the use of broth micro-dilution method (Table 7).

**Susceptibility of non-*Candida albicans*** **to single-plant extracts, extract combinations and conventional drugs**

All the four non-*C. albicans* strains (*C. glabrata* ATCC 2950, *C. glabrata* VVc 004, *C. tropicalis* ATCC 750 and *C. tropicalis* 0210) were susceptible to a total of 5 plant extracts including extract combinations and Amphotericin B drug, of which the aq. extract (24.4 °C) of *M. rubrostipulata (*ZOI: 16.22 ± 0.55- 38.33 ± 0.17) demonstrated significantly highest zones of inhibition. The other antimicrobial agents with moderate ZOIs in decreasing order included: Amphotericin B, methanol extract of *K. anthotheca*, combination of aq. extract (24.4 °C) of *M. rubrostipulata* + aq. extract (24.4 °C) of *K. anthotheca,* and aq. extract (60 °C) of *D. dissectus* + methanol extract of *K. anthotheca,* among others as shown in Table 6.

All the four *C. glabrata* ATCC 2950, *C. glabrata* VVc 004, *C. tropicalis* ATCC 750 and *C. tropicalis* 0210 were not susceptible to fluconazole pharmaceutical grades. Among the tested non-*C. albicans* species, only *C. tropicalis* 0210 was susceptible to fluconazole analytical grade (Table 6), which justified the use of broth micro-dilution method (Table 7).

**Susceptibility of both** ***C. albicans*** **and non-*C. albicans*** **species to plant extracts/combinations and conventional drugs**

All the *C. albicans*, *C. glabrata* and *C. tropicalis* species were susceptible to 2 single-plant extracts, one extract combination and one conventional drug, of which *M. rubrostipulata* aq.(24.4 °C)* (*ZOI: 18.00 ± 1.00 to 38.33 ± 0.17) demonstrated significantly the highest zones of inhibition. The other extracts, extract combinations and conventional drug with moderate-to-low ZOIs in decreasing order include: Amphotericin B (ZOI: 11.98 ± 0.11 to 22.32 ± 0.32), combination of aq. extract (60 °C) of *D. dissectus* + methanol extract of *K. anthotheca* (ZOI: 7.89 ± 0.26 to 19.67 ± 0.37), and methanol extract of K. *anthotheca* (ZOI: 10.11 ± 0.31 to 15.11 ± 0.65), respectively (Table 6).

**Table 6:** ZOI of single extracts, extract combinations, extract diluent (DMSO) and conventional drugs on selected *Candida* species

Antimicrobial agents	*Candida* species/strains	*Candida albicans*				Non-*Candida albicans*			
		Resistant to fluconazole		Susceptible to fluconazole		Susceptible to fluconazole			
	Extracts and their combinations	*C. albicans* ATCC 10231	*C. albicans* 0796	*C. albicans* ATCC 90028	*C. albicans* 0770a	*C. glabrata* ATCC 2950	*C. glabrata* VVc 004	*C. tropicalis* ATCC 750	*C. tropicalis* 0210
*M. rubrostipulata* (Mr)	Mr aqueous^1^	18.44 ± 1.20^a^	19.00 ± 2.54^a^	20.56 ± 0.56^a^	18.00 ± 1.00^aj^	20.78 ± 1.04^a^	16.22 ± 0.55^a^	38.33 ± 0.17^a^	19.22 ± 0.46^a^
	Mr aqueous^3^	7.89 ± 0.20^b^	8.33 ± 0.37^b^	8.78 ± 1.12^b^	7.22 ± 0.15^b^	18.00 ± 0.58^b^	0.00 ± 0.00^b^	14.44 ± 0.58^b^	0.00 ± 0.00^b^
	Mr Methanol	10.11 ± 0.42^cb^	11.44 ± 0.41^cb^	11.22 ± 0.49^cb^	11.11 ± 0.35^c^	0.00 ± 0.00^c^	0.00 ± 0.00^cb^	8.11 ± 0.31^c^	0.00 ± 0.00^cb^
	Mr aqueous^1^ + Mr Methanol	0.00^d^	12.89 ± 0.99^dc^	14.22 ± 1.39^di^	0.00 ± 0.00^d^	0.00 ± 0.00^dc^	9.11 ± 0.48^djl^	22.44 ± 0.73^d^	10.56 ± 0.24^dj^
	Mr aqueous^1^ + Mr aqueous^3^	12.78 ± 0.60^cejk^	0.00 ± 0.00^e^	0.00 ± 0.00^e^	0.00 ± 0.00^d^	0.00 ± 0.00^ec^	0.00 ± 0.00^eb^	22.00 ± 0.50^ed^	0.00 ± 0.00^eb^
	Mr aqueous^3^ + Mr methanol	11.11 ± 1.31^cf^	9.56 ± 0.41^fbkd^	11.33 ± 0.75^fbl^	11.44 ± 0.38^fc^	0.00 ± 0.00^fc^	0.00 ± 0.00^fb^	13.44 ± 0.24^fb^	0.00 ± 0.00^fb^
	Mr methanol + Mr aqueous^3^ + Mr aqueous^1^	14.67 ± 0.93^gj^	10.22 ± 0.94^gbc^	0.00 ± 0.00^ge^	0.00 ± 0.00^d^	0.00 ± 0.00^gc^	0.00 ± 0.00^gb^	21.33 ± 0.37^gdu^	0.00 ± 0.00^gb^
	Mr aqueous^1^ + Mr Methanol + Mr aqueous^3^	10.33 ± 1.01^bch^	12.11 ± 0.99^hcf^	15.67 ± 1.05^hi^	0.00 ± 0.00^d^	8.67 ± 0.44^hj^	9.11 ± 0.45^hjl^	18.78 ± 0.22^hikp^	0.00 ± 0.00^hb^
*K. anthotheca* (Ka)	Ka aqueous^1^	11.33 ± 0.65^ci^	13.78 ± 0.85^ic^	15.22 ± 0.22^i^	13.78 ± 0.46^ic^	0.00 ± 0.00^ic^	0.00 ± 0.00^ib^	19.22 ± 0.40^i^	8.33 ± 0.53^i^
	Ka methanol	15.00 ± 1.05^j^	13.22 ± 0.62^jc^	15.00 ± 0.67^ji^	15.11 ± 0.65^jai^	10.33 ± 0.37^j^	10.11 ± 0.31^j^	12.56 ± 0.69^jb^	10.89 ± 0.11^j^
	Ka aqueous^1^ + Ka Methanol	13.33 ± 1.09^kijlfp^	12.11 ± 0.61^kc^	17.33 ± 0.47^ki^	10.22 ± 0.92^kbc^	0.00 ± 0.00^kc^	0.00 ± 0.00^ kb^	17.11 ± 0.35^kl^	7.78 ± 0.28^ki^
*D. dissectus* (Dd)	Dd aqueous^2^	11.33 ± 0.41^ cl^	10.89 ± 0.63^lbc^	11.44 ± 0.29^lc^	11.00 ± 0.24^lc^	0.00 ± 0.00^lc^	8.78 ± 0.36^ l^	15.22 ± 0.49^ lb^	7.67 ± 0.24^li^
*M. rubrostipulata* (Mr) + *K. anthotheca* (Ka)	Mr aqueous^1^ + Ka methanol	13.89 ± 1.06^mijl^	23.44 ± 0.58^ m^	0.00 ± 0.00^me^	0.00 ± 0.00^d^	24.33 ± 1.13^ m^	19.33 ± 0.58^ m^	28.00 ± 0.69^ m^	0.00 ± 0.00^mb^
	Ka aqueous^1^ + Mr aqueous^1^	0.00^nd^	19.33 ± 1.33^na^	19.56 ± 0.58^nak^	0.00 ± 0.00^d^	11.56 ± 0.44^nj^	10.22 ± 0.52^nj^	22.11 ± 0.84^nd^	10.44 ± 0.24^nj^
*D. dissectus* (Dd) + *M. rubrostipulata* (Mr)	Dd aqueous^2^ + Mr aqueous^1^	10.33 ± 0.17^cobk^	11.44 ± 0.67^obc^	0.00 ± 0.00^oe^	0.00 ± 0.00^d^	0.00 ± 0.00^oc^	0.00 ± 0.00^ob^	21.11 ± 0.45^oid^	0.00 ± 0.00^ob^
D. dissectus (Dd) + *K. anthotheca* (Ka)	Dd aqueous^2^ + Ka aqueous^1^	10.89 ± 1.69^cp^	15.33 ± 0.71^pcq^	13.00 ± 1.03^pcif^	11.56 ± 1.02^pc^	0.00 ± 0.00^pc^	0.00 ± 0.00^pb^	16.78 ± 0.70^pl^	0.00 ± 0.00^pb^
	Dd aqueous^2^ + Ka methanol	19.22 ± 0.40^a^	16.00 ± 0.17^uac^	15.00 ± 0.47^ui^	14.89 ± 0.31^ui^	11.56 ± 1.21^uj^	7.89 ± 0.26^ul^	19.67 ± 0.37^ui^	12.56 ± 0.34^u^
*D. dissectus* (Dd) + *K. anthotheca* (Ka) + *M. rubrostipulata* (Mr)	Dd aqueous^2^ + Ka aqueous^1^ + Mr aqueous^1^	0.00^qd^	18.11 ± 1.74^qa^	0.00 ± 0.00^qe^	0.00 ± 0.00^d^	0.00 ± 0.00^qc^	0.00 ± 0.00^qb^	22.22 ± 0.78^qd^	8.11 ± 0.35^qi^
	Dd aqueous^2^ + Ka methanol + Mr aqueous^1^	19.33 ± 0.37^a^	20.22 ± 0.68^vam^	12.89 ± 1.17^vcif^	0.00 ± 0.00^d^	20.56 ± 0.63^va^	9.56 ± 0.58^vjl^	23.89 ± 0.45^vd^	8.00 ± 0.50^vi^
Controls (Ctrls) (n = 57)	Fluconazole analytical grade (+ ve ctrl)	0.00^rd^	0.00 ± 0.00^re^	0.00 ± 0.00^re^	0.00 ± 0.00^d^	0.00 ± 0.00^rc^	0.00 ± 0.00^rb^	0.00 ± 0.00^r^	32.37 ± 0.15^r^
	Fluconazole pharmaceutical grade (+ ve ctrl)	0.00^sd^	0.00 ± 0.00^se^	0.00 ± 0.00^se^	0.00 ± 0.00^d^	0.00 ± 0.00^sc^	0.00 ± 0.00^sb^	0.00 ± 0.00^sr^	0.00 ± 0.00^sb^
	Amp B (+ ve ctrl)	20.00 ± 0.11^a^	22.32 ± 0.32^wm^	21.16 ± 0.23^wa^	13.42 ± 0.54^wci^	19.11 ± 0.11^wb^	11.98 ± 0.11^w^	17.74 ± 0.21^wikp^	18.25 ± 0.12^w^
	DMSO_80% (-ve ctrl)	0.00 ± 0.00^td^	0.00 ± 0.00^te^	0.00 ± 0.00^te^	0.00 ± 0.00^d^	0.00 ± 0.00^tc^	0.00 ± 0.00^tb^	0.00 ± 0.00^tr^	0.00 ± 0.00^tb^
ANOVA (F)		466.56	332.93	613.68	226.95	1203.35	1215.23	1403.56	4440.59
*p*-value		0.00	0.00	0.00	0.00	0.00	0.00	0.00	0.00

Key: Aqueous^1^ =  cold water at 24.4 °C; aqueous^2^ =  hot water at 60 °C; aqueous^3^ =  hot water at 87 °C; ZOI =  zone of inhibition (mean ± SEM (mm), n = 9); DMSO =  dimethyl sulfoxide; Amp B = Amphotericin B

Mean comparisons of different extracts and conventional drugs (controls) were performed by one-way ANOVA followed by Tukey’s HSD post hoc multiple comparison test

Mean values with different superscript letter codes in the same column signifies significant difference (p < 0.05), similar letter codes signify no significant difference (p > 0.05)

#### Minimum inhibitory concentrations (MICs) and minimum fungicidal concentrations (MFCs) of the antimicrobial agents against tested *C. albicans* and non-*C. albicans species*

**Antifungal activity of single-plant extracts, extracts combinations and conventional drugs against** ***C. albicans*** **species (MICs/MFCs)**

Fluconazole analytical grade was the most active among the conventional drugs used, since it was resistant to only *C. albicans* 0796. However, fluconazole pharmaceutical grade and amphotericin B showed resistance to at least two *Candida albicans* strains, with all being resisted by *C. albicans* 0796 (Table 7).

Ten plant extracts and extract combinations showed antifungal activity to all the susceptible and resistant strains of *C. albicans* including *C. albicans* 0796 which resisted all the conventional drugs. Out of the ten active plant extracts and extracts combinations, five demonstrated very high antifungal activities (very low MICs), in decreasing antifungal strengths they included: combination of aq. extract (87 °C) of *M. rubrostipulata* + methanol extracts of *M. rubrostipulata* (MIC: 0.39 ± 0.00 to 3.13 ± 0.00; MFC: ≥ 50); methanol extract of *M. rubrostipulata* (MIC: 0.78 ± 0.00 to 3.13 ± 1.56; MFC: 12.50±0.00-100.00±0.00); methanol extract of *K. anthotheca* (MIC: 1.56 ± 0.00 to 6.25 ± 0.00; MFC: 12.50 ± 0.00 to 50.00 ± 0.00); aq. extract (24.4 °C) of *M. rubrostipulata* (MIC: 3.13 ± 0.00 to 6.25 ± 0.00; MFC: 12.50 ± 0.00 to 50.00 ± 0.00); and combination of aq. extract (60 °C) of *D. dissectus* + methanol extracts of *K. anthotheca* (MIC: 0.78 ± 0.00 to 12.50 ± 0.00; MFC: 12.500 ± 0.00 to 100.00 ± 0.00), respectively.

All the ten active plant extracts and extract combinations tested on the *C. albicans* strains, demonstrated fungistatic effect to the microbial organism, of which, the lowest MFCs were registered by aq. extract (24.4 °C) of *M. rubrostipulata* (MFC: 12.50 ± 0.00 to 50.00 ± 0.00) and methanol extract of *K. anthotheca* (MFC: 12.50 ± 0.00 to 50.00 ± 0.00), and the highest MFC was shown by aq. extract (87 °C) of *M. rubrostipulata* (MFC: > 50 to > 200).

***Antifungal activity of antimicrobial agents (single-plant extracts, extracts combinations and conventional drugs) against non-C. albicans*** **species (MICs/MFCs)**

Fluconazole analytical grade demonstrated the greatest antifungal activity to all the non-*C. albicans* species, while fluconazole pharmaceutical grade and amphotericin B showed resistance to at least two non-*C. albicans* species (Table 7). Five plant extracts and extract combinations showed antifungal activity to all the susceptible and resistant strains of *C. glabrata* and *C. tropicalis* (Table 7). The highest antifungal activity (lowest MIC) was exhibited by methanol extract of *K. anthotheca* (MIC: 1.04 ± 0.26 to 12.50 ± 0.00; MIC: 25.000 ± 0.000 to 100.00 ± 0.000), followed by aq. extract (24.4 °C) of *M. rubrostipulata*, combinations of aq. extract (24.4 °C) of *M. rubrostipulata* + aq. extract (24.4 °C) of *K. anthotheca,* and aq. extract (60 °C) of *D. dissectus* + methanol extract of *K. anthotheca*, respectively, among others (Table 7).

All the five plant extracts and extract combinations that showed antifungal activity to all the susceptible and resistant strains of non-*C. albicans* species demonstrated fungistatic effect to the microbial organism, of which, the lowest MFCs were registered by methanol extract of *K. anthotheca* (MFC: 25.000 ± 0.000 to 100.00 ± 0.00) across the test organisms (Table 7).

**Antifungal activity of single-plant extracts, extracts combinations and conventional drugs across both** ***C. albicans*** **and non-*C. albicans*** **species**

Three plant extracts and extract combinations exhibited antifungal activity across all the tested *C. albicans*, *C. glabrata* and *C. tropicalis* species, the most active with the lowest MIC was methanol extract of *K. anthotheca* (MIC: 1.04 ± 0.26 to 12.50 ± 0.00; MFC: 12.50 ± 0.00 to 100.00 ± 0.000) followed by aq. extract (24.4 °C) of *M. rubrostipulata* (MIC: 3.13 ± 0.00 to 20.83 ± 4.17; MFC: 12.50 ± 0.00 to 200.00 ± 0.00) and least was aq. extract (60 °C) of *D. dissectus* + methanol extract of *K. anthotheca* methanol (MIC: 0.78 ± 0.00 to 50.00 ± 0.00), respectively (Table 7).

None of the conventional drugs used (fluconazole analytical grade, fluconazole pharmaceutical grade and amphotericin B) showed antifungal activity across all the tested *C. albicans*, *C. glabrata* and *C. tropicalis* species (Table 7).

**Table 7:**
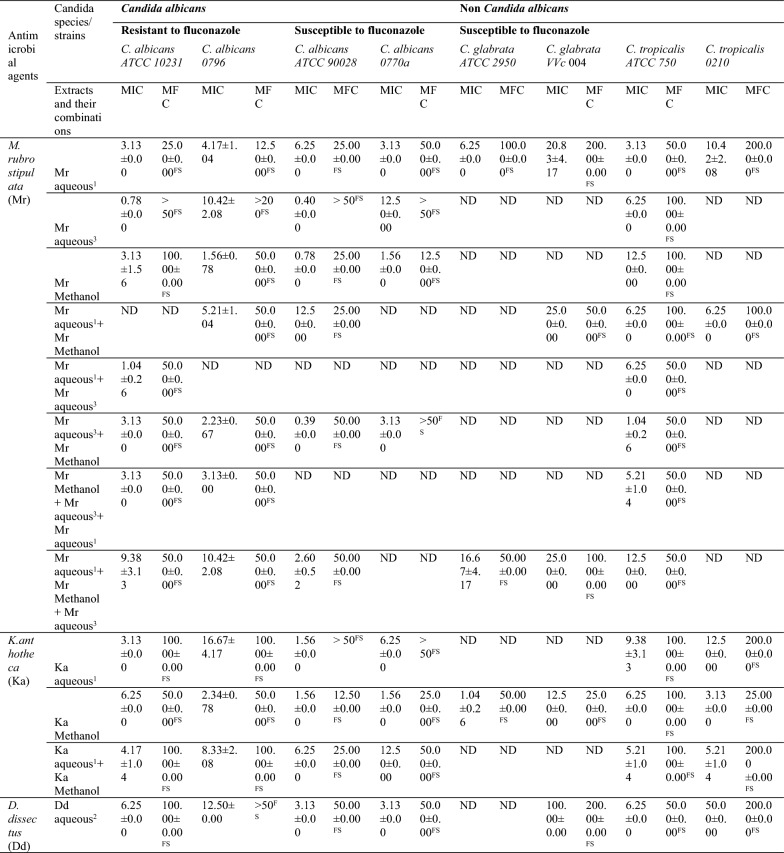

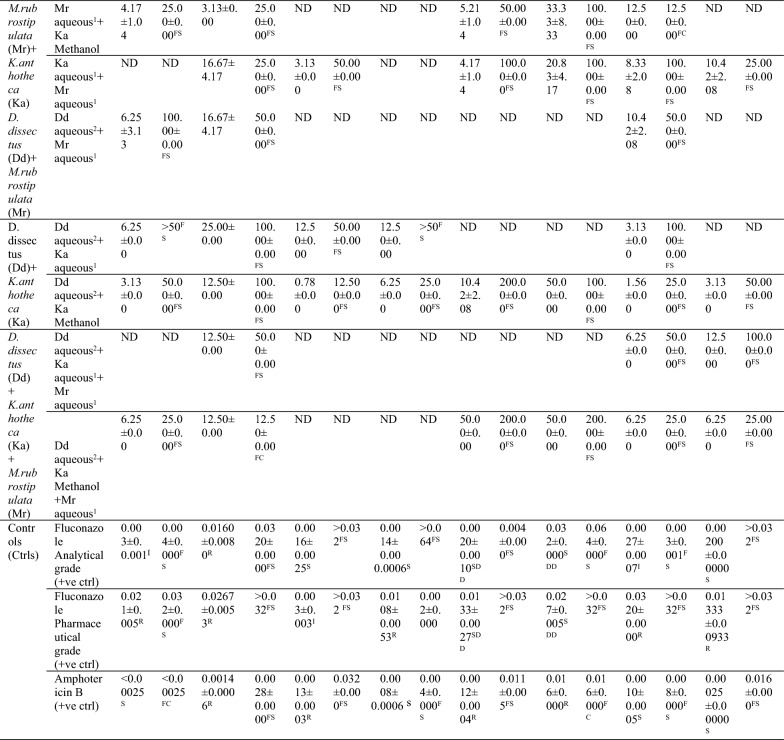
Antifungal activity (MIC/MFC) of single extracts, extracts combinations and conventional drugs against *Candida* species

Key: Aqueous^1^ =  cold water at 24.4 °C; aqueous^2^ =  hot water at 60 °C; aqueous^3^ =  hot water at 87 °C; S-susceptible; SSD-susceptible dose dependent; R-resistant; ND =  not determined (extracts and extracts combinations that did not show antifungal activity at preliminary screening stage/agar well diffusion)

Values of MIC and MFC were expressed as mean ± SEM (mg/ml), n = 3)

Susceptibility of *Candida * species to antifungal drugs (controls) were interpreted as: S-susceptible, I-intermediate, SSD-susceptible dose dependent, R-resistant. Efficacy of MFC were interpreted as: MIC < MFC—fungistatic (FS); MIC = MFC—fungicidal (FC)

## Discussion

### Plant extracts yields in different solvents and screening of their antifungal activity

Antifungal potentials and plant extract yields significantly varied in the five solvents (petroleum ether, methanol, aqueous (aq.) at 24.4 °C, aq. at 60 °C and aq. at 87 °C), with aq. solvent (87 °C) demonstrating the greatest extraction yields.

The variations in each of the plant extract yields across the five solvents with diverse antifungal potentials, can be used in selection of proper extraction solvents based on the type, polarity and bioactivity of the phytochemicals in the different plant extracts [41]. Although the methanol extracts of *K. anthotheca* and aq. extracts (24.4 °C) of *M. rubrostipulata* resulted in moderate yields of polar bioactive molecules, they exhibited significant antifungal activities across both resistant and susceptible strains of tested *C. albicans, C. glabrata* and *C. tropicalis* species. This showed that methanol and aq. extracts at 24.4 °C are suitable solvents for recovery of antifungal constituents from *K. anthotheca* and *M****.**** rubrostipulata*, respectively. The best solvent for extraction of *D. dissectus* was aq. (60 °C) since it was the only extract of *D. dissectus* that showed antifungal activity. Petroleum ether was not suitable solvent for extraction of any of the tested plants since all its extracts were inactive to all the tested* Candida* species. These findings are similar to that of Suurbaar et al. [42] in which methanol and aq. extracts of *Ricinus communis* exhibited higher anti-candida activity than petroleum ether extracts.

Important to note was that, although the aq. solvent (87 °C) had the greatest extraction yields, they demonstrated lower antifungal potentials than the aq. extracts (24.4 °C). The low activity could be explained by volatilization of some of the active antifungal compounds due to high temperatures. A study by Moomin et al. [43] found out that, other than the polarity of the extraction solvent, the yields and composition of bioactive constituents in the plant extracts are also reliant on temperature. In their study, cold water extract contained more bioactive metabolites like flavonoids, than hot water extracts. This was associated with metabolism or loss of thermo-unstable phytocompounds responsible for antimicrobial activity. However all extracts of *M. foetida* and *S. dawei* failed to exhibit any anti-candida activity across all tested strains of *C. albicans*, *C. glabrata* and *C. tropicalis* species at the screening phase, despite being the most preferred plants by the community of Pader district for treatment of candidiasis by the local communities and herbalists [13]. This could possibly suggest that the plant species are either not efficacious or that acute OPC and VVC could be associated with other microbial conditions that need to be scientifically verified. This is because a study by Bedore and Geinoro [44] in Ethiopia, reported that the crude methanol roots extracts of *M. foetida* exhibited good antibacterial activity against *Streptococcus agalactiae*. There is thus, likelihood that acute OPC/VVC is associated with other conditions like bacterial infections that are treated by *M. foetida*. *M. foetida* is widely used to treat opportunistic infections among people living with HIV/AIDS in Uganda especially fever, cough and diarrhoea [45]. Bukenya-Ziraba and Kamoga [46] reported the rhizome formulations of *S. dawei* to be used for treatment of diarrhoea in poultry in Jinja, Eastern Uganda. An ethnobotanical survey by Akwongo et al. [13] in Pader district, Northern Uganda reported diarrhoea in humans as one of the signs of acute OPC, thus indicating that *S. dawei* might also treat other conditions associated with OPC/VVC.

### Antifungal activity of antimicrobial agents (single-plant extracts, extracts combinations and conventional drugs) to *Candida* species

#### Susceptibility of *C. albicans* and non-*C. albicans* to antimicrobial agents

The fluconazole drugs were fungistatic to all the microbial agents, with fluconazole analytical grade being the most active antifungal conventional drug. Likewise, single extracts of *K. anthotheca* methanol and *M****.**** rubrostipulata* (aq. at 24.4 °C), and extracts combination of *D. dissectus* (aq. at 60 °C) + *K. anthotheca* methanol exhibited broad-spectrum antifungal activities against both susceptible and resistant strains of *C. albicans*, *C. glabrata* and *C. tropicalis* species, with the best antifungal activity being demonstrated by methanol stem bark extract of *K. anthotheca.*

Although fluconazole analytical grade did not show any ZOI against all tested *C. albicans*, *C. glabrata* and *C. tropicalis* species, its MIC demonstrated the greatest antifungal activity compared to fluconazole pharmaceutical grade and amphotericin B. The wide use of azoles like fluconazole as a first-line drug for many fungal infections can be attributed to their antifungal efficacy [47]. According to Bhattacharya et al. [48], fluconazole like most clinically available antifungals, inhibits fungal growth by targeting one of the essential enzymes of fungus [14 α-demethylase (Erg11p)] used in ergosterol biosynthesis. Ergosterol is responsible for regulation of membrane permeability and fluidity of the fungal cells. Fluconazole binds with the enzyme 14 α-demethylase (Erg11p) and inhibits its ergosterol pathways, thus, leading to production of toxic sterol (14α methylergosta 8-24 (28) dienol) that can inhibit fungal growth.

However, the fungistatic nature of fluconazole to* Candida* species, means that, the treatment of candida infections can easily lead to acquired resistance in the case of drug overuse [49]. In this study, fluconazole analytical grade showed zero zone of inhibition against *C. albicans*, *C. glabrata* and *C. tropicalis* indicating some level of resistance to fluconazole. This was demonstrated by antifungal inactivity of fluconazole to clinical resistant isolates of *Candida albicans* 0796. This is in agreement with a study conducted by Dhasarathan et al. [50] in which only 5.88% of tested *Candida* species were susceptible to fluconazole, and all clinical isolates of *C. albicans* resisted fluconazole drugs. The clinical isolates of *C. albicans* are highly resistant to fluconazole including pharmaceutical grades due to their evolutionary adaptation during the struggle to survive azole treatments, which has led to widespread antifungal drug resistance [50]. Bhattacharya et al. [48] noted many cases of point mutations in fungal ERG11 enzymes isolated from resistant clinical isolates, the mutations lower the fluconazole binding in the active sites of the ERG11p enzymes. Silva-Beltran et al. [51] noted increased fungal tolerance to the mechanisms of actions of traditional conventional antifungal drugs.

Similarly in this study; non-*Candida albicans* tested viz, *C. glabrata* and *C. tropicalis* showed some level of antifungal resistance to fluconazole analytical grade, by demonstrating susceptible dose dependence to both control and clinical strains of *C. glabrata* ATCC 2950 and *C. glabrata* VVc 004 isolates, respectively; and intermediate dose to *C. tropicalis* ATCC 750. Resistance of antifungal drugs to non-*C. albicans* species has been reported by many studies. For instance, a study conducted by Uno et al. [52] among immunosuppressed patients in Nigeria, found out that *C. glabrata* showed multidrug resistance to wide range of antifungals tested including fluconazole and amphotericin B. Likewise, Silva et al. [53] detected point mutations in *ERG11* gene that was responsible for drug resistance in the clinical *C. glabrata* isolates (C108G, C423T and A1581G). This worrying trend of resistance of non-*C. albicans* to available antifungal drugs has also been noted by Valand and Girija [54], thus, the urgency to discover new broad spectrum antifungal treatment options to curb emerging cases of antifungal drug resistance. However, amphotericin B significantly exhibiting wide ZOI to all *C. albicans* than fluconazole, could be due to fungicidal nature of amphotericin B, which binds to ergosterol in the pathogenic fungal cell membrane and form pores that causes monovalent ions like Cl^−^, H^+^, K^+^ and Na^+^ to rapidly leak out of the cells leading to cell death [48]. Despite the susceptibility of all *C. albicans* species to amphotericin B shown by the ZOI, its MIC showing resistance to a half of the* Candida* species tested shows that it is not the anti-candida drug of choice. This confirms argument of Bhattacharya et al. [48], that amphotericin B is not often required for treatment of invasive candidiasis. This finding has shown that, antifungal activity of antifungal agents can best be determined by use of MICs than ZOI. This is because various factors like composition of the medium, duration and temperature of the diffusion phase before incubation, among others, influences the zone of inhibition [55].

The combination of aq. extract (87 °C) of *M. rubrostipulata* + methanol extract of *M. rubrostipulata*, and single methanol extract of *M. rubrostipulata* demonstrating the highest antifungal activities against all the tested *candida albicans* species, with moderate ZOI also shows that the antifungal potential of these plants are best determined using MICs than agar well diffusion, due to inability of aq. agar matrix to permit diffusion of some antimicrobial compounds due to difference in polarities [55]. The high antifungal activity of these extracts against all tested *C. albicans* could be due to presence of antifungal bioactive compounds that justifies their traditional use. Stangeland et al. [56] screened the phytochemicals in water and methanol extracts of *M. rubrostipulata* and found that it contained alkaloids, flavonoids, saponins and also terpenoids [57], which were responsible for its various antimicrobial activity. For instance, Ahmad et al. [58] and Huang et al. [59] indicated that terpenoids antifungal activity against *C. albicans* is through inhibition of membrane protein H^+^-ATPase in the fungal cell membrane responsible for maintaining electrochemical proton gradient across the fungal cell membrane, for the intracellular pH regulation and nutrients uptake for cell growth; thus its inhibition leads to intracellular acidification and death of the fungal cells.

The methanol stem bark extract of *K. anthotheca* exhibited the best broad spectrum antifungal activities against both susceptible and resistant *C. albicans, C. glabrata* and *C. tropicalis* species more than all the conventional drugs used. This could be attributed to presence of more bioactive compounds of intermediate polarity with broad spectrum anti-candida activity, which justified methanol as a better extraction solvent. A systematic review on Khaya species by Olatunji et al. [60] showed that, unique to genus Khaya (Meliaceae) including *K.* *anthotheca* is that they contains limonoids as one of the secondary metabolites with great antimicrobial significance. Therefore, the broad spectrum antifungal activity of methanol extract of *K.* *anthotheca* could also be attributed to different types of bioactive limonoids it contains. Olatunji et al. [60] stated that limonoids (highly specialized oxygenated triterpenoids) offer great antimicrobial activities of these plants to promote good health. Limonoids destroys the fungal cell wall and cell membrane leading to exudation of intracellular substances that affect the growth and metabolism of the pathogenic organism [61]. Limonoids also exert their antimicrobial effect by inhibition of biofilm formation as well as cell-to-cell signalling mechanism [62]. Thus, the methanol extract of *K. anthotheca* contains lead antifungal limonoids that could be isolated for new antifungal drug development.

Combinations of aq. extract (60 °C) of *D. dissectus* + methanol extract of *K. anthotheca* exhibited third best broad-spectrum antifungal activities against both susceptible and resistant *C. albicans*, *C. glabrata* and *C. tropicalis* species tested*.* This could be linked to the curative antifungal phytochemical constituents that these extracts contain. In addition to the phytochemicals of *K. anthotheca* discussed above, *D. dissectus* contains mainly resin glycosides in addition to tropane, alkaloids, phenolic compounds, coumarins, sesquiterpenoid and flavonoids including isoflavones [63]. Glycosides exert their antifungal activity through forming complex with sterols in the fungal cell membranes. This results in pore formations in the membrane leading to its ruptures and loss in membrane integrity, consequently fungal cell death [64]. Most of these phytochemicals with antifungal activity were isolated from the roots of plants belonging to genus *Merremia (*now called *Distimake*), and are known to be therapeutically relevant including antifungal properties [63] that justifies their traditional use. This makes *D. dissectus* a good candidate plant for more antifungal drug development if more researches are done on it. According to Silva-Beltran et al. [51], the antifungal efficacy of medicinal plants are due to ability of the phytochemicals to damage the fungal cell membrane and cell wall by interfering with fungal ATP synthesis, calcium and potassium ions flow, respectively. From this study, medicinal plants *K. anthotheca, M. rubrostipulata and D. dissectus* are therefore promising plant candidates for new antifungal drugs since their extracts generally contain various bioactive compounds like limonoids, terpenoids, alkaloid, glycosides, carbohydrates, sesquiterpenoid and polyphenols which are selective inhibitors of fungal cell wall glucan biosynthesis and cell membrane ergosterol. This finding is supported by Jin [65] who stated that, antifungals that targets destruction of the fungal cell wall and ergosterol in the cell membrane are promising candidate for new antifungal drugs development. This is because the targeted vital components of fungal cell wall (glucans, chitin and glycoproteins) are absent in the humans. Also, humans have cholesterol in the place of ergosterol in fungal cell membranes, a vital structural component responsible for fluidity.

Although *C. albicans* accounts for 80 to 90% of fungal infections [66], non-*C. albicans* species have also become a big problem due to their reduced susceptibility to available antifungal drugs, and have become predominant fungal pathogens of many clinical types of candidiasis [67]. Also Turner and Butler [68] reported that, although *C. albicans* is the leading cause of fungal infections, together with other four non-*C. albicans* species (*C. glabrata, C. tropicalis, C. parapsilosis,* and *C. krusei)* they account for about 90% of overall fungal infections. Thus, antimicrobial agents with antifungal potency against all *Candida albicans* and non-*C. albicans* species would be the best antifungal option. From this study therefore, methanol extracts of *K. anthotheca*, aq. extract (24.4 °C) of *M. rubrostipulata* and combination of aq. extract (60 °C) of *D. dissectus* + methanol extract of *K. anthotheca* posed the best antifungal activity since they acted on both clinical and resistant strains of all* Candida* species used in this study.

#### Fungicidal/fungistatic effect of antimicrobial agents on *Candida* species

All plant extracts that demonstrated great antifungal activity across all *C. albicans* (combination of aq. extract (87 °C) of *M. rubrostipulata* + methanol extract of *M. rubrostipulata,* and methanol extract of *M. rubrostipulata*), across both *C. glabrata* and *C. tropicalis* species (combination of aq. extract (24.4 °C) of *M. rubrostipulata* + aq. extract (24.4 °C) of *K. anthotheca*), and across all *C. albicans*, *C. glabrata* and *C. tropicalis* species (methanol extract of *K. anthotheca*, aqueous extract (24.4 °C) of *M. rubrostipulata*, and combination of aq. extract (60 °C) of *D. dissectus* + methanol extract of *K. anthotheca*) were all fungistatic to the tested* Candida* species. This could be due to their ability to inhibit fungal growth. Hawser and Islam [69] stated that, the less fungicidal agents like azoles, exert their antifungal activity through inhibition of budding process of pathogenic fungi. Kumar et al. [70] found out that, although fungicidal agents used for treatment of invasive candidiasis result in probability of early cure and decreased probability of fungal infection recurrence, improvement in patients’ survival still remains a big challenge. Also a study by Vendetti et al. [71] on paediatric candidaemia in USA, found no significant difference in patients’ mortality between those treated with fungistatic and fungicidal drugs. They then concluded that, both fungicidal and fungistatic agents can be used as definitive therapy for treatment of candidiasis. Since both fungicidal (amphotericin B) and fungistatic (fluconazole) conventional drugs used in this study were not effective against some resistant strains of* Candida* species, it means that the fungistatic plant remedies that showed high antifungal effectiveness against all* Candida* species in this study could provide better treatment option for candidiasis.

The emerging cases of antimicrobial resistance calls for urgent need to find alternative drugs to treat both susceptible and resistant *C. albicans* and non-*C. albicans* species*.*

### Limitations of the study

Slight variability in the thickness of the agar layer and diffusion rate of the antifungal agents has effects on the diameters of ZOI. This could have led to inconsistency in the correlation between ZOIs and MICs.

## Conclusion

Multidrug resistant candida strains such as *C. albicans* 0796, is a big threat to treatment of fungal infections, since it showed resistance to fluconazole (analytical and pharmaceutical grades) and amphotericin B. Thus, for the tested* Candida* species, aq. extract (24.4 °C) of *M. rubrostipulata*, methanol extract of *Khaya anthotheca,* and combination of aq. extract (60 °C) of *D. dissectus* + methanol extract of *K. anthotheca* proved more effective in the treatment of candidiasis, they demonstrated broad spectrum antifungal activities against 8 strains of resistant and susceptible *C. albicans, C. glabrata and C. tropicalis* species than fluconazole and amphotericin B drugs. All the broad-spectrum antifungal plant extracts exhibited fungistatic effect to the tested* Candida* species, demonstrating their ability to inhibit fungal growth. Hence, these extracts can offer better treatment option for candidiasis if they are standardized and also their active curative compounds isolated and made into antifungal drugs.

## Data Availability

All data generated and analysed during this study have been included in this manuscript.

## Abbreviations

OPC: Oropharyngeal candidiasis
VVC: Vulvovaginal candidiasis
DMSO: Dimethyl sulfoxide
ATCC: American Type Culture Collection
MIC: Minimum inhibitory concentration
MFC: Minimum fungicidal concentration
FIC: Informant Consensus Factor
GPS: Global Positioning System
POWO: Plants of the World
BHIB: Brain heart infusion broth
MHA: Mueller–Hinton agar medium
SDA: Sabouraud dextrose agar
CLSI: Clinical Laboratory Institute
EUCAST: European Committee on Antimicrobial Susceptibility Testing
RPMI: Roswell Park Memorial Institute
ZOI: Zone of inhibition
MOPs: 3-(N-Morpholino) propane sulfonic acid
HCl: Hydrochloric acid
SPSS: Statistical Package for the Social Sciences
ANOVA: Analysis of variance
Tukey’s HSD: Tukey’s honest significant difference test
SEM: Standard error of the mean

## References

[1] Bongomin F, Gago S, Oladele RO, Denning DW. Global and multi-national prevalence of fungal diseases—estimate precision. J fungi. 2017;3(4):57. 10.3390/jof3040057 PMC5753159 29371573

[2] Parkes-Ratanshi R, Achan B, Kwizera R, Kambugu A, Meya D, Denning DW. Cryptococcal disease and the burden of other fungal diseases in Uganda; Where are the knowledge gaps and how can we fill them? Mycoses. 2015;58:85–93. 26449512 10.1111/myc.12387

[3] Bongomin F, Kwizera R, Namusobya M, van Rhijn N, Andia-Biraro I, Kirenga BJ, et al. Re-estimation of the burden of serious fungal diseases in Uganda. Ther Adv Infect Dis. 2024;11:20499361241228344. 10.1177/20499361241228345 PMC10848809 38328511

[4] Rubaihayo J, Tumwesigye NM, Konde-Lule J, Wamani H, Nakku-Joloba E, Makumbi F. Frequency and distribution patterns of opportunistic infections associated with HIV/AIDS in Uganda. BMC Res Notes. 2016;9(1):1–16. 27927247 10.1186/s13104-016-2317-7 PMC5142427

[5] McKeny PT, Nessel TA, Zito PM. Antifungal antibiotics. 2019. 30844195

[6] Pathadka S, Yan VKC, Neoh CF, Al-Badriyeh D, Kong DCM, Slavin MA, et al. Global consumption trend of antifungal agents in humans from 2008 to 2018: data from 65 middle-and high-income countries. Drugs. 2022;82(11):1193–205. 35960433 10.1007/s40265-022-01751-x PMC9402496

[7] Kakudidi E, Anywar G, Fredrick A, Jasper O-O. Antifungal Medicinal Plants Used by Communities Adjacent to Bwindi Impenetrable National Park, South-Western Uganda. Eur J Med Plants. 2015;7:184–92.

[8] Berman J, Krysan DJ. Drug resistance and tolerance in fungi. Nat Rev Microbiol. 2020;18(6):319–31. 32047294 10.1038/s41579-019-0322-2 PMC7231573

[9] Denning DW. Antifungal drug resistance: an update. Eur J Hosp Pharm. 2022;29(2):109–12. 35190454 10.1136/ejhpharm-2020-002604 PMC8899664

[10] Abdallah EM, Alhatlani BY, de Paula MR, Martins CHG. Back to Nature: medicinal plants as promising sources for antibacterial drugs in the post-antibiotic era. Plants. 2023;12(17):3077. 37687324 10.3390/plants12173077 PMC10490416

[11] Navarro V, Villarreal ML, Rojas G, Lozoya X. Antimicrobial evaluation of some plants used in Mexican traditional medicine for the treatment of infectious diseases. J Ethnopharmacol. 1996;53(3):143–7. 8887021 10.1016/0378-8741(96)01429-8

[12] Murtaza G, Mukhtar M, Sarfraz A. A review: antifungal potentials of medicinal plants. J Bioresour Manag. 2015;2(2):4.

[13] Akwongo B, Katuura E, Nsubuga AM, Tugume P, Andama M, Anywar G, et al. Ethnobotanical study of medicinal plants utilized in the management of candidiasis in Northern Uganda. Trop Med Health. 2022;50(1):78. 36242066 10.1186/s41182-022-00471-y PMC9569084

[14] Svetaz L, Zuljan F, Derita M, Petenatti E, Tamayo G, Caceres A, et al. Value of the ethnomedical information for the discovery of plants with antifungal properties. A survey among seven Latin American countries. J Ethnopharmacol. 2010;127(1):137–58. 19782744 10.1016/j.jep.2009.09.034

[15] Vaghasiya Y, Chanda S. Screening of methanol and acetone extracts of fourteen Indian medicinal plants for antimicrobial activity. Turkish J Biol. 2007;31(4):243–8.

[16] Oloya B, Namukobe J, Ssengooba W, Afayoa M, Byamukama R. Phytochemical screening, antimycobacterial activity and acute toxicity of crude extracts of selected medicinal plant species used locally in the treatment of tuberculosis in Uganda. Trop Med Health. 2022;50(1):1–13. 35177126 10.1186/s41182-022-00406-7 PMC8851836

[17] Abubakar AR, Haque M. Preparation of medicinal plants: basic extraction and fractionation procedures for experimental purposes. J Pharm Bioallied Sci. 2020;12(1):1. 32801594 10.4103/jpbs.JPBS_175_19 PMC7398001

[18] Aruwa CE, Amoo S, Kudanga T. Phenolic compound profile and biological activities of Southern African *Opuntia**ficus*-indica fruit pulp and peels. Lwt. 2019;111:337–44.

[19] Khoram Z, Naine A, Rafieinezha R, Hakimaneh SM, Hakimaneh SMR, Shayehg SS, et al. The antifungal effects of two herbal essences in comparison with nystatin on the Candida strains isolated from the edentulous patients. J Contemp Dent Pract. 2019;20(6):716. 31358715

[20] CLSI M27-A3. Clinical and and Laboratory Standards Institute. Reference method for broth dilution antifungal susceptibility testing of yeasts. Approved Standard third edition, M27-A3. Wayne: CLSI M27-A3; 2008.

[21] EUCAST. Method for the determination of minimum inhibitory concentration (MIC) by broth dilution of fermentative yeasts. Clin Microbiol Infect. 2003;9(8):i–viii.

[22] Berkow EL, Lockhart SR, Ostrosky-Zeichner L. Antifungal susceptibility testing: current approaches. Clin Microbiol Rev. 2020;33(3):e00069-e19. 32349998 10.1128/CMR.00069-19 PMC7194854

[23] CLSI M27-A2. Reference method for broth dilution antifungal susceptibility testing of yeasts, approved standard. CLSI Doc M27-A2. 2002.

[24] Nejad BS, Rajabi M, Mamoudabadi AZ, Zarrin M. In vitro anti-candida activity of the hydroalcoholic extracts of heracleum persicum fruit against phatogenic* Candida* species. Jundishapur J Microbiol. 2014;7(1): e8703. 25147655 10.5812/jjm.8703 PMC4138661

[25] Wenji KY, Rukmi I, Suprihadi A. In vitro antifungal activity of methanolic and chloroform mint leaves (*Mentha**piperita* L.) Extracts against *Candida**albicans*. J Phys Conf Ser. 2019;1217: 012136.

[26] Ruiz-Duran J, Torres R, Stashenko EE, Ortiz C. Antifungal and antibiofilm activity of Colombian essential oils against different Candida strains. Antibiotics. 2023;12(4):668. 37107030 10.3390/antibiotics12040668 PMC10135359

[27] Kebede B, Shibeshi W. In vitro antibacterial and antifungal activities of extracts and fractions of leaves of *Ricinus**communis* Linn against selected pathogens. Vet Med Sci. 2022;8(4):1802–15. 35182460 10.1002/vms3.772 PMC9297757

[28] Rex JH. Method for antifungal disk diffusion susceptibility testing of yeasts: approved guideline. Clin Lab Stand Inst. 2009.

[29] Bhat V, Sharma SM, Shetty V, Shastry CS, Rao CV, Shenoy S, et al. Characterization of herbal antifungal agent, *Origanum**vulgare* against oral Candida spp. isolated from patients with Candida-Associated denture stomatitis: an In vitro study. Contemp Clin Dent. 2018;9(1):S3. 29962756 10.4103/ccd.ccd_537_17 PMC6006875

[30] Balouiri M, Sadiki M, Ibnsouda SK. Methods for in vitro evaluating antimicrobial activity: a review. J Pharm Anal. 2016;6(2):71–9. 29403965 10.1016/j.jpha.2015.11.005 PMC5762448

[31] Arenas RJB, Villanueva RMD, Simbahan JF, Obusan MCM. Antimicrobial activity of endophytic and rhizospheric fungi associated with soft Fern (*Christella* sp.) and cinderella weed (*Synedrella**nodiflora*) inhabiting a hot spring in Los Baños, Laguna, Philippines. Acta Med Philipp. 2022;56(10):e1417.

[32] Vaou N, Stavropoulou E, Voidarou C, Tsakris Z, Rozos G, Tsigalou C, et al. Interactions between medical plant-derived bioactive compounds: focus on antimicrobial combination effects. Antibiotics. 2022;11(8):1014. 36009883 10.3390/antibiotics11081014 PMC9404952

[33] Odongo EA, Mutai PC, Amugune BK, Mungai NN, Akinyi MO, Kimondo J. Evaluation of the antibacterial activity of selected Kenyan medicinal plant extract combinations against clinically important bacteria. BMC Complement Med Ther. 2023;23(1):100. 37013533 10.1186/s12906-023-03939-4 PMC10069043

[34] Desrini S, Girardot M, Imbert C, Mustofa M, Nuryastuti T. Screening antibiofilm activity of invasive plants growing at the Slope Merapi Mountain, Central Java, against *Candida**albicans*. BMC Complement Med Ther. 2023;23(1):232. 37438777 10.1186/s12906-023-04044-2 PMC10339508

[35] Scorzoni L, Benaducci T, Almeida AMF, Silva DHS, da Bolzani VS, Gianinni MJ. The use of standard methodology for determination of antifungal activity of natural products against medical yeasts Candida sp. and Cryptococcus sp. Braz J Microbiol. 2007;38(3):391–7.

[36] Ohikhena FU, Wintola OA, Afolayan AJ. Evaluation of the antibacterial and antifungal properties of *Phragmanthera**capitata* (Sprengel) Balle (Loranthaceae), a mistletoe growing on rubber tree, using the dilution techniques. Sci World J. 2017;2017:1. 10.1155/2017/9658598 PMC5470015 28642934

[37] O’brien J, Wilson I, Orton T, Pognan F. Investigation of the *Alamar**blue* (resazurin) fluorescent dye for the assessment of mammalian cell cytotoxicity. Eur J Biochem. 2000;267(17):5421–6. 10951200 10.1046/j.1432-1327.2000.01606.x

[38] van de Sande WWJ. In vitro susceptibility testing for black grain eumycetoma causative agents. Trans R Soc Trop Med Hyg. 2021;115(4):343–54. 33537781 10.1093/trstmh/traa184 PMC8046409

[39] Tmmedia. TMH 105-potato dextrose agar (as per USP/BP/EP/JP/IP). Titan Biotech Ltd. 2020.

[40] Nguni TL, dos Santos Abrantes PM, McArthur C, Klaasen JA, Fielding BC. Evaluation of synergistic anticandidal activity of Galenia africana extract and fluconazole against Candida albicans and Candida glabrata. J Herb Med. 2022;32: 100503.

[41] El Mannoubi I. Impact of different solvents on extraction yield, phenolic composition, in vitro antioxidant and antibacterial activities of deseeded *Opuntia**stricta* fruit. J Umm Al-Qura Univ Appl Sci. 2023;9:1–9.

[42] Suurbaar J, Mosobil R, Donkor AM. Antibacterial and antifungal activities and phytochemical profile of leaf extract from different extractants of *Ricinus**communis* against selected pathogens. BMC Res Notes. 2017;10(1):1. 29191226 10.1186/s13104-017-3001-2 PMC5709865

[43] Moomin A, Russell WR, Knott RM, Scobbie L, Mensah KB, Adu-Gyamfi PKT, et al. Season, storage and extraction method impact on the phytochemical profile of *Terminalia**ivorensis*. BMC Plant Biol. 2023;23(1):1–18. 36964494 10.1186/s12870-023-04144-8 PMC10039578

[44] Bedore B, Geinoro T. An In-Vitro antibacterial effect of *Momordica**foetida* and *Croton**macrostachyus* on *Streptococcus**agalactiae* Isolated from bovine mastitis. Int J Vet Sci Anim Husb. 2018;3(6):19–23.

[45] Anywar G, Kakudidi E, Byamukama R, Mukonzo J, Schubert A, Oryem-Origa H. Indigenous traditional knowledge of medicinal plants used by herbalists in treating opportunistic infections among people living with HIV/AIDS in Uganda. J Ethnopharmacol. 2020;246: 112205. 31476442 10.1016/j.jep.2019.112205

[46] Bukenya-Ziraba R, Kamoga D. An inventory of medicinal plants used in treating poultry diseases in Jinja district, eastern Uganda. Afr J Ecol. 2007;45:31–8.

[47] Gintjee TJ, Donnelley MA, Thompson GR III. Aspiring antifungals: review of current antifungal pipeline developments. J Fungi. 2020;6(1):28. 10.3390/jof6010028 PMC7151215 32106450

[48] Bhattacharya S, Sae-Tia S, Fries BC. Candidiasis and mechanisms of antifungal resistance. Antibiotics. 2020;9(6):312. 32526921 10.3390/antibiotics9060312 PMC7345657

[49] Berkow EL, Lockhart SR. Fluconazole resistance in* Candida* species: a current perspective. Infect Drug Resist. 2017;10:237–45. 28814889 10.2147/IDR.S118892 PMC5546770

[50] Dhasarathan P, AlSalhi MS, Devanesan S, Subbiah J, Ranjitsingh AJA, Binsalah M, et al. Drug resistance in *Candida**albicans* isolates and related changes in the structural domain of Mdr1 protein. J Infect Public Health. 2021;14(12):1848–53. 34794907 10.1016/j.jiph.2021.11.002

[51] Silva-Beltran NP, Boon SA, Ijaz MK, McKinney J, Gerba CP. Antifungal activity and mechanism of action of natural product derivates as potential environmental disinfectants. J Ind Microbiol Biotechnol. 2023;50(1):kuad036. 37951298 10.1093/jimb/kuad036 PMC10710307

[52] Uno UU-U, Upla DA, Okoli MO, Dominic ON. Molecular characterization and biofilm formation of* Candida* species isolated from immunosuppressed HIV seropositive individuals in Calabar metropolis, Nigeria. Int Res J Innov Eng Technol. 2023;7(9):1.

[53] dos Silva DB, Rodrigues LMC, de Almeida AA, de Oliveira KMP. Novel point mutations in the ERG11 gene in clinical isolates of azole resistant* Candida* species. Mem Inst Oswaldo Cruz. 2016;111:192–9. 26982177 10.1590/0074-02760150400 PMC4804502

[54] Valand N, Girija UV. Candida pathogenicity and interplay with the immune system. Microb Pathog Infect Immun. 2021. 10.1007/978-3-030-67452-6_11. 10.1007/978-3-030-67452-6_11 34661898

[55] Eloff JN. Avoiding pitfalls in determining antimicrobial activity of plant extracts and publishing the results. BMC Complement Altern Med. 2019;19(1):1–8. 31113428 10.1186/s12906-019-2519-3 PMC6530048

[56] Stangeland T, Wangensteen H, Katuura E, Lye KA, Paulsen BS. Antioxidant and anti-plasmodial activity of extracts from three Ugandan medicinal plants. 2010.

[57] Alamgir ANM. Secondary metabolites: Secondary metabolic products consisting of C and H; C, H, and O; N, S, and P elements; and O/N heterocycles. Progr Drug Res. 2018;74:165–309.

[58] Ahmad A, Khan A, Yousuf S, Khan LA, Manzoor N. Proton translocating ATPase mediated fungicidal activity of eugenol and thymol. Fitoterapia. 2010;81(8):1157–62. 20659536 10.1016/j.fitote.2010.07.020

[59] Huang W, Wang Y, Tian W, Cui X, Tu P, Li J, et al. Biosynthesis investigations of terpenoid, alkaloid, and flavonoid antimicrobial agents derived from medicinal plants. Antibiotics. 2022. 10.3390/antibiotics11101380. 36290037 10.3390/antibiotics11101380 PMC9598646

[60] Olatunji TL, Odebunmi CA, Adetunji AE. Biological activities of limonoids in the Genus Khaya (Meliaceae): a review. Futur J Pharm Sci. 2021;7(1):1–16.

[61] Wang H, Zeng X, Feng W, Yu L, Zhai W, Bai W, et al. Antifungal activity and mechanism of limonoids from lemon peel against Penicillium. Food Ferment Ind. 2019;45(5):75–9.

[62] Shi Y-S, Zhang Y, Li H-T, Wu C-H, El-Seedi HR, Ye W-K, et al. Limonoids from Citrus: chemistry, anti-tumor potential, and other bioactivities. J Funct Foods. 2020;75: 104213.

[63] Olatunji TL, Adetunji AE, Olisah C, Idris OA, Saliu OD, Siebert F. Research progression of the genus merremia: a comprehensive review on the nutritional value, ethnomedicinal uses, phytochemistry, pharmacology, and toxicity. Plants. 2021;10(10):2070. 34685875 10.3390/plants10102070 PMC8537340

[64] Khan H, Khan Z, Amin S, Mabkhot YN, Mubarak MS, Hadda TB, et al. Plant bioactive molecules bearing glycosides as lead compounds for the treatment of fungal infection: a review. Biomed Pharmacother. 2017;93:498–509. 28675856 10.1016/j.biopha.2017.06.077

[65] Jin Y-S. Recent advances in natural antifungal flavonoids and their derivatives. Bioorg Med Chem Lett. 2019;29(19): 126589. 31427220 10.1016/j.bmcl.2019.07.048

[66] Talapko J, Juzbašić M, Matijević T, Pustijanac E, Bekić S, Kotris I, et al. *Candida**albicans*—the virulence factors and clinical manifestations of infection. J Fungi. 2021;7(2):79. 10.3390/jof7020079 PMC7912069 33499276

[67] Deorukhkar SC, Saini S, Mathew S. Non-albicans Candida infection: an emerging threat. Interdiscip Perspect Infect Dis. 2014;2014:615958. 25404942 10.1155/2014/615958 PMC4227454

[68] Turner SA, Butler G. The Candida pathogenic species complex. Cold Spring Harb Perspect Med. 2014;4(9): a019778. 25183855 10.1101/cshperspect.a019778 PMC4143104

[69] Hawser S, Islam K. Comparisons of the effects of fungicidal and fungistatic antifungal agents on the morphogenetic transformation of *Candida**albicans*. J Antimicrob Chemother. 1999;43(3):411–3. 10223599 10.1093/jac/43.3.411

[70] Kumar A, Zarychanski R, Pisipati A, Kumar A, Kethireddy S, Bow EJ. Fungicidal versus fungistatic therapy of invasive Candida infection in non-neutropenic adults: a meta-analysis. Mycology. 2018;9(2):116–28. 30123667 10.1080/21501203.2017.1421592 PMC6059084

[71] Vendetti N, Bryan M, Zaoutis TE, Damianos A, Fisher BT. Comparative effectiveness of fungicidal vs fungistatic therapies for the treatment of paediatric candidaemia. Mycoses. 2016;59(3):173–8. 26692326 10.1111/myc.12449

